# Phylogenetic Relationships between Four *Salix* L. Species Based on DArT Markers

**DOI:** 10.3390/ijms141224113

**Published:** 2013-12-11

**Authors:** Jerzy A. Przyborowski, Paweł Sulima, Anna Kuszewska, Dariusz Załuski, Andrzej Kilian

**Affiliations:** 1 Departament of Plant Breeding and Seed Production, University of Warmia and Mazury, Plac Łódzki 3, Olsztyn 10-724, Poland; E-Mails: jerzy.przyborowski@uwm.edu.pl (J.A.P.); anna.kuszewska@uwm.edu.pl (A.K.); dariusz.zaluski@uwm.edu.pl (D.Z.); 2 Diversity Arrays Technology Pty Limited, 1 Wilf Crane Crescent, Yarralumla ACT 2600, Australia; E-Mail: a.kilian@diversityarrays.com

**Keywords:** phylogenetic relationships, genetic diversity, species identity, diversity arrays technology, DArT, *Salix*, willow

## Abstract

The objectives of this study were to evaluate the usefulness of DArT markers in genotypic identification of willow species and describe genetic relationships between four willow species: *Salix viminalis*, *S. purpurea*, *S. alba* and *S. triandra*. The experimental plant material comprised 53 willow genotypes of these four species, which are popularly grown in Poland. DArT markers seem to identify *Salix* species with a high degree of accuracy. As a result, the examined species were divided into four distinct groups which corresponded to the four analyzed species. In our study, we observed that *S. triandra* was very different genetically from the other species, including *S. alba* which is generally classified into the same subgenus of *Salix.* The above corroborates the findings of other authors who relied on molecular methods to reveal that the classification of *S. triandra* to the subgenus *Salix* was erroneous. The Principal Coordinate Analysis (PCoA) and the neighbor-joining dendrogram also confirmed the clear division of the studied willow genotypes into four clusters corresponding to individual species. This confirmed the usefulness of DArT markers in taxonomic analyses and identification of willow species.

## 1. Introduction

According to Argus [1], the genus *Salix* L. (willow) comprises around 450 species and an undefined number of natural and artificial hybrids. Willows are characterized by a high degree of genetic diversity. The genus inhabits mostly cold and moderate climate zones of the northern hemisphere [2,3]. Selected species are grown for a wide range of practical applications, including in the power engineering sector, pharmaceutical industry, wicker production and environmental management [4–8]. In recent years, willow has attracted considerable interest from farmers, industrial manufacturers and scientists. This popularity gave rise to numerous research publications investigating the genus *Salix* L. [6–12]. New willow farms are created, and willow biomass has a variety of industrial applications. *Salix purpurea* and *S. alba* plants are used in pharmaceutical applications [5,7], whereas *S. viminalis* and *S. triandra* belong to species used for energy production [6,8,9].

**Figure 1.**
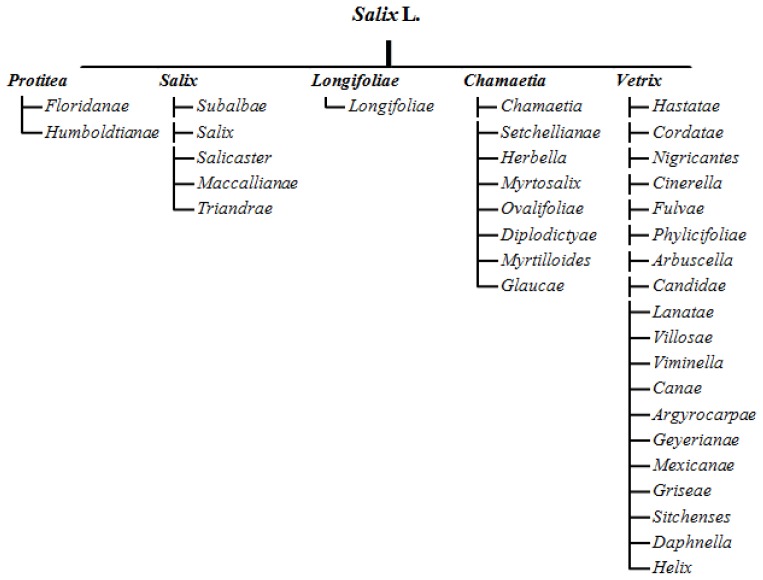
Systematic of genus *Salix* L. divided into five subgenera [1].

Since the Linnaean era [13], the attempts to systematize the genus *Salix* have been fraught with many problems, and the willow’s taxonomic status remains a subject of debate among botanists [1,2,13–17]. Argus [1] distinguished five basic subgenera of the genus *Salix: Protitea*, *Salix*, *Longifoliae*, *Chamaetia*, *Vetrix* (Figure 1). The main problem in systematic classification stems from willow’s ability to produce a wide variety of interspecies hybrids. The willow is also characterized by high seasonal, ecological and environmental variation. Sub-genera and species of *Salix* L. are generally identified based on morphological traits, including the color, shape and hairiness of leaves and shoots, as well as the structure, shape and color of inflorescences [2,3]. The fact that the willow is a dioecious genus with different flowering and leaf formation rates further complicates the matter. In many species, the pigmentation of leaves and shoots changes during growth and under the influence of environmental factors, such as temperature and day length. For this reason, the issue remains open despite many years of research attempting to systematize the taxon’s classification status [1,2,16,17].

Molecular techniques play an increasingly important role in taxonomic studies and analyses of genetic diversity of living organisms [18–21]. Techniques that rely on DNA analysis are a useful analytical tool in every developmental stage of living organisms. Those methods are not affected by environmental factors; they are characterized by high repeatability and ease of use. Species identification with molecular markers is particularly recommended when morphological analyses produce ambiguous or problematic results [18,20]. The above applies to members of the genus *Salix*. Molecular methods for species identification rely these days on analyses of DNA polymorphism [19–21]. Taxonomic classification is based on similarities or differences between the genetic material of the studied forms. The determination of species-specific sequences of DNA supports correct identification. Molecular methods support the identification of organisms at developmental stages that are difficult to determine with the use of conventional methods, most of which rely on evaluations of morphological and anatomical traits [20,22,23].

Various marker systems capable of generating several (Random Amplification of Polymorphic DNA-RAPD, Inter-Simple Sequence Repeat-ISSR) to thousands of markers (Single Nucleotide Polymorphism-SNP) have been developed in recent decades [23–26]. Researchers have a preference for low-cost systems that effectively identify a large number of molecular markers within a relatively short period of time. Diversity Arrays Technology (DArT) [27] is such a system. By relying on DNA microarrays, this technique supports simultaneous analyses of thousands of loci distributed across the entire genome without prior knowledge of its sequence [28–31]. To date, DArT markers have supported the genomic analysis of over 70 organisms (http://www.diversityarrays.com, [32]). DArT is used mainly for genome mapping and sequencing, QTL (Quantitative Trait Loci) identification, marker-assisted selection, genetic diversity analyses and studies of varietal and species diversity of organisms. The DArT technique is not affected by the genome size or the ploidy level of the analyzed organisms [33].

The objectives of this study were: (1) to develop DArT for willow; (2) to evaluate the usefulness of DArT markers in genotyping of willow species; and (3) to describe genetic relationships between four willow species which are popularly grown in Poland, and in other European and world regions.

## 2. Results and Discussion

The analysis of 53 willow genotypes (Table 1) on DArT discovery array resulted in identification of 1362 markers with 95% average call rate and average technical reproducibility derived from full technical replication of all samples used in analysis at 99.9%. An analysis of basic genetic differentiation parameters (values of Φ*_ST_* and Nei’s genetic distance index-*D_S_*) (Table 2) confirmed the results of other studies which suggest that the genus *Salix* is characterized by considerable genetic diversity [17,34,35]. In our study, the average value of Φ*_ST_* reached 0.754 for only four analyzed species, which indicates a high diversity among them (Table 3).

*S. triandra* was highly genetically different from the other species, including *S. alba* which is generally classified into the same subgenus of *Salix* [1,2,16]. The above corroborates the findings of other authors who relied on molecular methods [17,34] to reveal that the classification of *S triandra* to the subgenus *Salix* was erroneous. Chen *et al*. [17] have argued that the section *Triandrae* should be excluded from the subgenus *Salix*. The above claim was formulated based on the results of molecular analyses, differences in bark exfoliation on old stems (which is more similar to *Chosenia*) [2], smaller amounts of nuclear DNA [36] and the fungal species *Melampsora amygdalinae* which causes willow rust and colonizes only *S. triandra* [37].

In the group of the analyzed species, the lowest genetic distance was observed between *S. purpurea* and *S. viminalis* (Table 2). It should be noted, however, that their genetic differentiation parameters (Φ*_ST_* = 0.665; *D_S_* = 0.264) were indicative of considerable differences. DArT markers seem to identify *Salix* species with a high degree of accuracy. Despite the presence of close phylogenetic relationships between *S. purpurea* and *S. viminalis* and the classification of both species to the subgenus *Vetrix* [1,2,16], the observed genetic differences between those species support the correct identification of the analyzed willow genotypes.

An analysis of genetic variation within the examined willow species revealed a low level of diversity (Table 4). The results of the analysis of molecular variance (AMOVA) confirmed the above observation. Only 25% of molecular variation was noted within species, whereas 75% was observed between the studied species (Table 3). The highest genetic variation within species was reported in *S. viminalis* (*I* = 0.126; *H_e_* = 0.084), and the lowest in *S. triandra* (*I* = 0.071; *H*e = 0.047).

Species-specific markers are valuable tools in analyses of species’ identities. In this study, a high number of private markers were determined for every examined species (Table 4). The highest number of species-specific markers was reported for *S. triandra* (135), which further asserts its distinctness from the remaining species. In practice, the effectiveness of species-specific markers increases with the number of analyzed genotypes originating from the largest possible group of species. The usefulness of species-specific DArT markers in analyses of other plant genera and species was confirmed by other authors [38,39].

**Figure 2.**
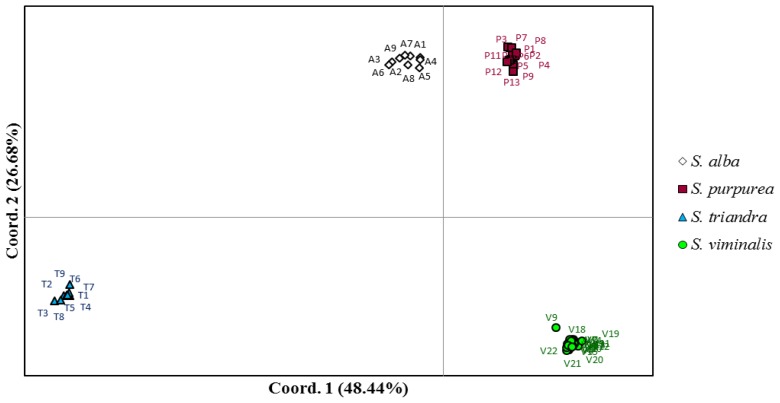
Principal coordinate analysis (PCoA) of 53 individual *Salix* plants based on DArT data.

**Figure 3.**
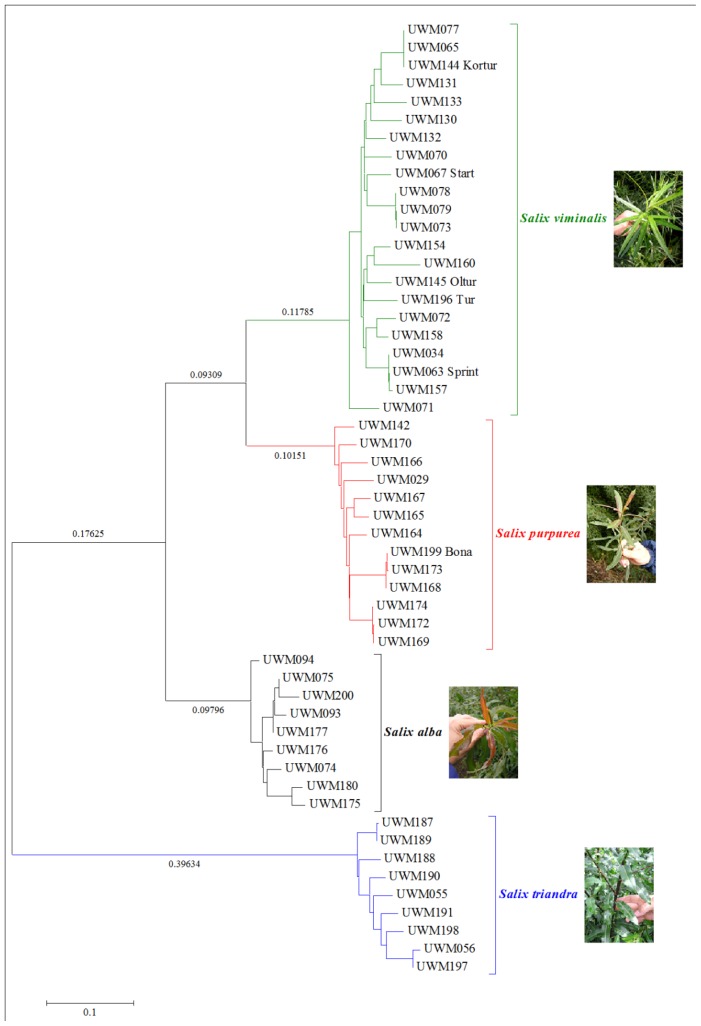
Neighbor-joining dendrogram showing the relationships among the all studied genotypes of *Salix* based on Nei’s genetic distances.

In our study, the Principal Coordinate Analysis (PCoA) explained 75.12% of genetic variation between willow species (Figure 2). As a result, the examined species were divided into four distinct groups of clusters which corresponded to the four analyzed species. Only the UWM071 genotype (identified as V9 in PCoA) appeared to be clearly separate from the *S. viminalis* group, which raises doubts about its taxonomic classification in the *S. viminalis* species. An evaluation of the morphological traits of the UWM071 genotype validated those doubts, and the genotype was ultimately classified as an interspecies hybrid of *S. viminalis* × *S. dasyclados*. The fact that the *S. purpurea* group was found in the proximity of *S. alba*, but it was significantly distant from the more related taxonomic group of *S. viminalis*, was also surprising. Despite the above, the structure of the neighbor-joining dendrogram points to greater genetic similarity between *S. purpurea* and *S. viminalis* (Figure 3). The dendrogram also confirmed the clear division of the studied willow genotypes into four clusters corresponding to individual species. A considerable genetic distance was also noted between *S. triandra* and the remaining species, which supports our previous observations concerning this taxon’s classification rank.

To date, molecular methods have been rarely used to analyze the species identity or taxonomic relationships within the genus *Salix* Leskinen and Alström-Rapaport [40] used ribosomal (5.8 S) ITS 1 and ITS 2 in a study investigating the phylogenesis of *Salicaceae* and phylogenetic relationships with *Flacourtiaceae*. Azuma, *et al*. [41] tested phylogenetic relationships of the genus *Salix* with the involvement of the chloroplast *rbcL* gene. Chen *et al*. [17] also relied on chloroplast markers (*rbcL* gene, *atpB-rbcL* and *trnD-T* spacers) to propose significant changes in the classification of the genus *Salix* Trybush *et al*. [34] confirmed the usefulness of amplified fragment length polymorphisms (AFLP) in genetic diversity analyses of a broad spectrum of willow species as well as in taxonomic studies.

## 3. Materials and Methods

### 3.1. Plant Material

The experimental material comprised 53 willow individuals from the collection of the Department of Plant Breeding and Seed Production at the University of Warmia and Mazury in Olsztyn, Poland. The analyzed genotypes were preliminarily classified into four species. *S. alba* was represented by nine individuals, *S. purpurea*-by 13, *S. triandra*-by 9 and *S. viminalis*-by 22 individuals (Table 1). The species were identified based on a botanical key [42].

### 3.2. DNA Isolation and DArT Protocol

The DNA of different genotypes was isolated from young leaf tissue according to the DNA Extraction Protocol for DArT (http://www.diversityarrays.com/sites/default/files/pub/DArT_DNA_isolation.pdf) [43].

### 3.3. Preparation of Genomic Representations

Genomic representations were generated by cutting 100 ng of a DNA samples from each used in study genotype of *Salix* with 2 units of both PstI and MspI restriction enzymes. A PstI adapter (5′-CAC GAT GGA TCC AGT GCA-3′ annealed with 5′-CTG GAT CCA TCG TGC A-3′) was ligated with T4 DNA ligase (NEB, Beverly, MA, USA). A 1 μL aliquot of the ligation product was used as a template in 50 μL amplification reactions with DArT-PstI primer (5′-GAT GGA TCC AGT GCA G-3′) and a PCR program applicable to all plant species tested so far: 94 °C for 1 min, followed by 30 cycles of 94 °C for 20 s, 58 °C for 40 s, 72 °C for 1 min, and 72 °C for 7 min.

### 3.4. Preparation of DArT Libraries and Arrays

Libraries of genomic representations were prepared essentially as by Jaccoud *et al*. [27]. Individual clones were grown in 384-well plates containing LB medium supplemented with 100 mg/L ampicillin and a “freezing mix” (1 × LB medium containing 4.4% glycerol, 8.21 g/L K_2_HPO_4_, 1.80 g/L KH_2_PO_4_, 0.50 g/L Na_3_-citrate, 0.10 g/L MgSO_4_ × 7 H_2_O, 0.90 g/L (NH_4_)_2_SO_4_, 100 mg/L Ampicilin and 100mg/L kanamycin). Small aliquots of the cultures were used as templates to amplify inserts according to Kilian *et al*. [33]. The quality of the amplifications was verified by gel electrophoresis, dried, dissolved in spotting buffer [33] and spotted in duplicate onto SuperChip poly-L-lysine slides (Erie Microarray, Portsmouth, NH, USA) by using aMicroGrid II arrayer (Biorobotics, Cambridge, UK). After printing, slides were heated to 80 °C for 2 h, incubated in hot water (95 °C) for 2 min, and dried by centrifugation.

### 3.5. Fingerprinting of DNA Samples

Genomic representations of individual *Salix* genotypes were generated by using the same complexity reduction method as the one used to generate the libraries. Genomic representations were concentrated 10-fold by precipitation with 1 vol of isopropanol, denatured and labeled with 1 mM Cy3-dUTP or 1 mM Cy5-dUTP and the exo-Klenow fragment of *Escherichia coli* DNA polymerase I (NEB) according to Kilian *et al*. [33]. Labeled representations, called targets, were added to 50 μL of a 50:5:1 mixture of ExpressHyb buffer (Clontech, Palo Alto, CA, USA), 10 g/L herring sperm DNA, and the FAM-labeled polylinker fragment of the plasmid used for library preparation as a reference [27]. The samples were denatured and hybridized to microarrays overnight at 65 °C. Slides were washed according to Jaccoud *et al*. [27] and scanned on an Affymetrix 428 (Santa Clara, CA, USA) or Tecan LS300 (Grödig, Salzburg, Austria) confocal laser scanner.

### 3.6. Image Analysis and Polymorphism Scoring

DArTsoft (Diversity Arrays Technology, Yarralumla, Australia) [44], a software package developed by DArT PL (http://www.diversityarrays.com/software.html) [44], was used to both identify and score the markers that were polymorphic within such an experiment. DArTsoft automatically localized the spots in all scanner image pairs generated in an experiment, rejected those with weak reference signals identified using the package of DArTsoft software: microarray image analysis, and computed and normalized background-subtracted relative hybridization intensities (calculated as log[cy3target/cy5reference]). The software then compared the relative intensity values for each individual genotype across slides by using a combination of fuzzy C-means clustering at a “fuzziness” level of 1.5 [27] and ANOVA: If two clusters (alleles) could be distinguished and the between-cluster variance in relative intensity was at least 80% of the total variance, the clone was called polymorphic and scored as 0 or 1. A clone was incorporated into the 0/1 scoring table of a particular experiment if it was scored with a probability of *p >* 0.95 in at least 90% of the slides (scoring probabilities were estimated by the clustering algorithm). Individual calls with *p <* 0.95 were scored as missing. Slides with <90% of the identified polymorphic markers scored at *p >* 0.95 were rejected (typically less than 5%).

### 3.7. Marker Scoring and Statistical Analysis

The results of polymorphic scoring are presented in an Excel binary file where “1” denotes the presence, and “0” the absence of a marker in genomic representation of a sample. The results were processed in the following applications: GenAlEx 6.5 [45,46], Aflpsurvey 1.0 [47], Popgene 1.32 [48] and MEGA 5.1 [49]. The basic genetic differentiation parameters were presented for the studied species. The number of observed alleles (*N*a), the number of effective alleles (*N*e) [50], the mean Nei’s gene diversity index (*H*e), the unbiased gene diversity index (*uH*e) [51], the Shannon diversity index (*I*) [52] and the number of private bands per species (*PrB*) were determined for the examined species. To assess the extent of genetic differentiation among species, were calculated pairwise distances (Φ*_ST_* values) according to Weir and Cockerham [53] and Nei’s genetic distances (*D_S_*) according to Nei [54]. An analysis of molecular variance (AMOVA) [55] was performed to determine the percentage share of components of genetic variation. Genetic variation of the studied willow genotypes was visualized by Principal Coordinate Analysis (PCoA) based on binary genetic distance (Euclidean distance). A neighbor-joining dendrogram [56] was developed based on Nei’s genetic distance matrix data [54].

## 4. Conclusions

Our findings contribute to scientific efforts aiming to standardize the taxonomic classification of the genus *Salix*, and they provide valuable knowledge about genetic relationships within and between the species of *S. alba*, *S. purpurea*, *S. triandra* and *S. viminalis*. The present study has confirmed the usefulness of DArT markers in taxonomic analyses and identification of willow species. Of great importance is the fact that willow species chosen for the research have major economic value in many countries. The biomass of *S. viminalis* and *S. triandra* is used on a large scale in the production of heat energy. As far as the pharmaceutical industry is concerned, of great significance is the bark of *S. purpurea* and *S. alba* that is used in the production of analgesic, antipyretic, anti-inflammatory and anti-rheumatic drugs. The obtained results will facilitate the assessment of genetic variation in *Salix* breeding materials. *Salix purpurea* and *S. alba* plants are used for the production of hybrids with an increased salicylic glycoside content in the bark (pharmaceutical applications) [57], whereas *S. viminalis* and *S. triandra* plants are included in breeding programs aiming to produce varieties with a high biomass energy potential [58].

## Figures and Tables

**Table 1.** List of 53 *Salix* genotypes used in this study, their name in collection and symbol in Principal Coordinate Analysis.

Species	Name in Collection	Symbol PCoA
*S. alba*	UWM075	A1
*S. alba*	UWM200	A2
*S. alba*	UWM180	A3
*S. alba*	UWM177	A4
*S. alba*	UWM176	A5
*S. alba*	UWM175	A6
*S. alba*	UWM074	A7
*S. alba*	UWM094	A8
*S. alba*	UWM093	A9
*S. purpurea*	UWM199	P1
*S. purpurea*	UWM174	P2
*S. purpurea*	UWM173	P3
*S. purpurea*	UWM172	P4
*S. purpurea*	UWM170	P5
*S. purpurea*	UWM169	P6
*S. purpurea*	UWM168	P7
*S. purpurea*	UWM167	P8
*S. purpurea*	UWM166	P9
*S. purpurea*	UWM165	P10
*S. purpurea*	UWM164	P11
*S. purpurea*	UWM029	P12
*S. purpurea*	UWM142	P13
*S. triandra*	UWM187	T1
*S. triandra*	UWM198	T2
*S. triandra*	UWM056	T3
*S. triandra*	UWM055	T4
*S. triandra*	UWM197	T5
*S. triandra*	UWM191	T6
*S. triandra*	UWM190	T7
*S. triandra*	UWM189	T8
*S. triandra*	UWM188	T9
*S. viminalis*	UWM079	V1
*S. viminalis*	UWM078	V2
*S. viminalis*	UWM077	V3
*S. viminalis*	UWM145	V4
*S. viminalis*	UWM154	V5
*S. viminalis*	UWM070	V6
*S. viminalis*	UWM073	V7
*S. viminalis*	UWM072	V8
*S. viminalis*	UWM071	V9
*S. viminalis*	UWM067	V10
*S. viminalis*	UWM065	V11
*S. viminalis*	UWM063	V12
*S. viminalis*	UWM034	V13
*S. viminalis*	UWM196	V14
*S. viminalis*	UWM160	V15
*S. viminalis*	UWM158	V16
*S. viminalis*	UWM157	V17
*S. viminalis*	UWM130	V18
*S. viminalis*	UWM144	V19
*S. viminalis*	UWM133	V20
*S. viminalis*	UWM132	V21
*S. viminalis*	UWM131	V22

**Table 2.** Matrix based on mean pairwise Φ*_ST_* distance (below diagonal) and matrix on Nei’s distance (*D_S_*) (above diagonal) between four species of *Salix*.

	*S. alba*	*S. purpurea*	*S. triandra*	*S. viminalis*
*S. alba*	–	0.320	0.687	0.371
*S. purpurea*	0.687	–	0.802	0.264
*S. triandra*	0.782	0.828	–	0.808
*S. viminalis*	0.707	0.665	0.822	–

**Table 3.** Analysis of molecular variance (AMOVA) based on 1362 DArT markers for 53 individuals in four species of *Salix* L. (*p* < 0.01).

Source of Variation	*d.f.*	Sum of Squares	Mean Squares	Estimated Variation	Total Variance	Φ*_ST_* (*p* < 0.01)
Among Species	3	8715.160	2905.053	225.787	75%	0.754
Within Species	49	3600.991	73.490	73.490	25%	
Total	52	12,316.151		299.276	100%	

**Table 4.** Genetic diversity parameters within each of the studied species of *Salix*.

Species	*N*	*N*a	*N*e	*I*	*H*e	*uH*e	*PrB*
*S. alba*	9	0.866	1.095	0.079	0.053	0.057	77
*S. purpurea*	13	0.829	1.114	0.097	0.065	0.068	37
*S. triandra*	9	0.647	1.081	0.071	0.047	0.050	135
*S. viminalis*	22	0.984	1.145	0.126	0.084	0.086	83
Mean	12.860	0.831	1.109	0.093	0.062	0.065	83

*N*: sample size; *N*a: number of different alleles; *N*e: mean number of effective alleles; *I*: Shannon’s diversity index; *H*e: expected Nei’s heterozygosity; *uH*e: unbiased expected Nei’s heterozygosity; *PrB*: number of private bands per species.
